# Single Nucleotide Variants (SNVs) of the Mesocorticolimbic System Associated with Cardiovascular Diseases and Type 2 Diabetes: A Systematic Review

**DOI:** 10.3390/genes15010109

**Published:** 2024-01-17

**Authors:** Mohammed Merzah, Shewaye Natae, János Sándor, Szilvia Fiatal

**Affiliations:** 1 Department of Public Health and Epidemiology, Faculty of Medicine, University of Debrecen, 4032 Debrecen, Hungary; mohammed.merzah@med.unideb.hu (M.M.);; 2 Doctoral School of Health Sciences, University of Debrecen, 4032 Debrecen, Hungary; 3 ELKH-DE Public Health Research Group, Department of Public Health and Epidemiology, Faculty of Medicine, University of Debrecen, 4032 Debrecen, Hungary

**Keywords:** CVDs, diabetes, gene variant, mesocorticolimbic, reward pathway, SNV, SNP

## Abstract

The mesocorticolimbic (MCL) system is crucial in developing risky health behaviors which lead to cardiovascular diseases (CVDs) and type 2 diabetes (T2D). Although there is some knowledge of the MCL system genes linked to CVDs and T2D, a comprehensive list is lacking, underscoring the significance of this review. This systematic review followed PRISMA guidelines and the Cochrane Handbook for Systematic Reviews of Interventions. The PubMed and Web of Science databases were searched intensively for articles related to the MCL system, single nucleotide variants (SNVs, formerly single nucleotide polymorphisms, SNPs), CVDs, T2D, and associated risk factors. Included studies had to involve a genotype with at least one MCL system gene (with an identified SNV) for all participants and the analysis of its link to CVDs, T2D, or associated risk factors. The quality assessment of the included studies was performed using the Q-Genie tool. The VEP and DAVID tools were used to annotate and interpret genetic variants and identify enriched pathways and gene ontology terms associated with the gene list. The review identified 77 articles that met the inclusion criteria. These articles provided information on 174 SNVs related to the MCL system that were linked to CVDs, T2D, or associated risk factors. The COMT gene was found to be significantly related to hypertension, dyslipidemia, insulin resistance, obesity, and drug abuse, with rs4680 being the most commonly reported variant. This systematic review found a strong association between the MCL system and the risk of developing CVDs and T2D, suggesting that identifying genetic variations related to this system could help with disease prevention and treatment strategies.

## 1. Introduction

Non-communicable diseases (NCDs) pose a significant global health challenge and are among the top causes of adult mortality worldwide [1]. In 2022, NCDs were estimated to account for 41 million (71%) of the 57 million global deaths, of which cardiovascular diseases (CVDs) caused 17.9 million (31%) of the global deaths and 44% of all deaths as a result of NCDs [1], whereas diabetes mellitus (DM) was attributed to 1.5 million (3%) of all global deaths and 4% of all NCD deaths [1]. Most NCDs share common risk factors, which are often categorized as behavioral or biological [2].

The mesocorticolimbic (MCL) system, originating in the ventral tegmental area (VTA) region of the brain [3], might play a crucial role in the development of key risky health behaviors leading to chronic NCDs of major public health importance. Studies have revealed that there is a strong association between the MCL system and the risk of developing CVDs [4,5]. A substantial body of research has demonstrated that certain single nucleotide variants (SNVs) of specific MCL genes are significant in the increased risk of CVDs. For instance, rs7396366, rs4680, and rs4714210 were found to be related to coronary artery disease [6]; rs4680 was associated with hypertension; rs4633 and rs4680 were linked to atherosclerosis [7]; and rs2097603, rs4633, rs4680, and rs174699 were associated with venous thrombosis [8]. Additionally, rs324420 was found to be related to an increased heart rate [9]. The mesolimbic system plays important roles in the regulation of behavior, vulnerability to stress, and drug abuse [10,11]. Stress is a potential activator of mesolimbic and mesocortical projections [12,13]. It is also associated with noticeable cardiovascular responses, like differential vasoconstrictor response, change in blood pressure, and heart rate [14,15]. The MCL system also regulates optimal cardiovascular responses such as the assimilation of sensory and behavioral information with cardiovascular homeostasis [4,14,16]. To sum up, it works as a connector between behaviors like locomotory and cognitive, and cardiovascular homeostasis, which result in CVDs [4,14].

Likewise, studies have revealed that the MCL system has some impacts on the etiology and pathogenesis of type 2 diabetes (T2D) and metabolic syndrome (MS) [17,18]. An animal experiment showed that increased dopamine tone in mesolimbic brain areas leads to an increased value of various rewarding stimuli, including food intake [19,20]. This fact may have determined an increased motivation for food consumption in the test animals, which at later stages, could result in obesity and deficits in glucose control [21].

Furthermore, environmental and genetic risk factors influence the incidence and severity of CVDs and T2D. Other behavioral risk factors that contribute to the development of CVDs and T2D are smoking, excessive alcohol intake, poor diet, drug addiction, and physical inactivity [22,23]. These lifestyle factors are closely linked to the MCL system, which involves a complex interplay between genetic and environmental influences. Research indicates that variations in MCL genes can increase susceptibility to CVDs and T2D among individuals with these risk factors [22,23]. Genome-wide association studies have revealed that heterogeneity can result in different susceptible genes being associated with CVDs and T2D [24,25].

Identifying genetic variants linked to the development of, or considered risk factors for, CVDs and T2D is critical for disease prevention and therapy. There is no comprehensive information from genetic association research on MCL system genes that have been identified as risk factors for CVDs and T2D. Therefore, this systematic review was undertaken to give a complete list of SNVs of the MCL system that are related to CVDs and T2D, as well as their possible risk factors.

## 2. Materials and Methods

### 2.1. Study Design and Search Strategy

**Figure 1:**
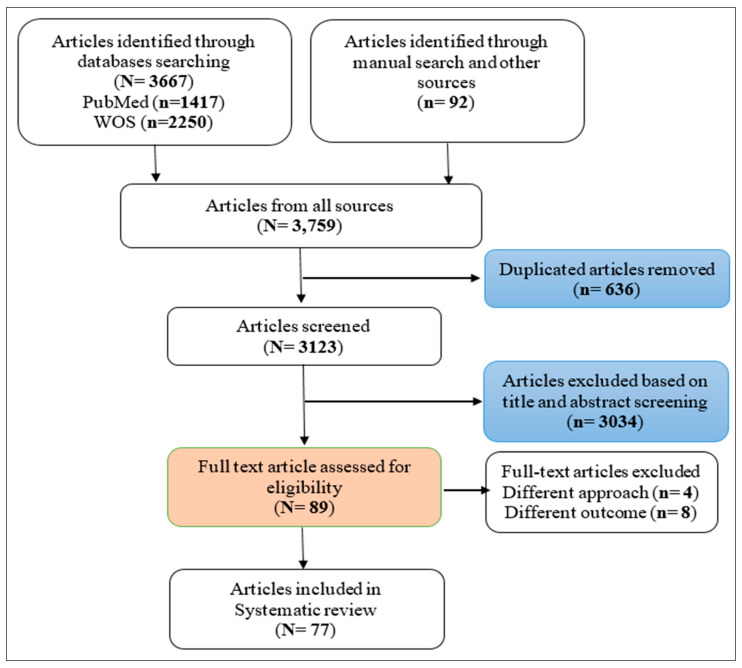
PRISMA flow chart of the included studies.

This review was conducted in accordance with PRISMA guidelines and the Cochrane Handbook for Systematic Reviews of Interventions [26]. Prior to sorting the studies for inclusion, the review protocol was registered in the international prospective register of systematic reviews, PROSPERO (ID: CRD42021273784). Two databases (PubMed and Web of Science) were searched intensively to identify articles that were related to the MCL system, SNPs, gene variants, and CVDs, T2D, or their risk factors. Those databases were used since they are considered the most fundamental sources of medical research. Search terms and keywords were developed based on the concepts that made up the research question by using the National Library of Medicine’s vocabulary thesaurus, MeSH, as indicated in Supplementary Tables S1–S3. To maximize our search sensitivity, the bibliographies of first hit articles, similar articles to those in PubMed, and articles in Google Scholar, ProQuest, and some related journals were manually screened to cover all published and unpublished related articles. The process of selecting studies is illustrated in Figure 1.

### 2.2. Inclusion Criteria

Studies published up to 31 May 2023 were included in this review based on the following criteria: (1) at least one gene (with an identified SNV) related to the MCL system was genotyped for all study participants; (2) the genes (with identified SNVs) were associated with CVDs, T2D, or their risk factors; and (3) primary studies were conducted in the English language and on humans only.

### 2.3. Exclusion Criteria

Studies must not have been conducted on psychiatric-related health statuses like schizophrenia or major depressive disorder (MDD). Furthermore, no limitation was created regarding the study type or characteristics of subjects.

### 2.4. Quality Assessment and Data Extraction

Quality assessment for all included studies was conducted using the standard genetic association study quality assessment tool (Q-Genie tool) [27]. Each article was evaluated on a scale of 1–77; the average score of all included articles was 71 (ranging from 52 to 77), which indicates good-quality studies (Supplementary Appendix S1). A preliminary synthesis of the extracted data from the included articles is indicated in Table 1. A thematic analysis was used since it is an appropriate method in the context of a systematic review of heterogeneous data [28]. Independently, two authors completed all of the above steps. In case of any inconsistency, the opinion and advice from a third reviewer was considered.

### 2.5. Bioinformatics Analysis

We performed a bioinformatics analysis to annotate and interpret genetic variants and to identify overrepresented biological functions and pathways associated with our identified genes and variant lists. The variant effect prediction (VEP) tool was used to annotate the functional effects of genetic variants [29]. The VEP tool was run with the human genome assembly GRCh38.p13 and the Ensembl transcript database release 109. For the functional annotation and enrichment analysis, the Database for Annotation, Visualization, and Integrated Discovery (DAVID) tools was used to identify enriched pathways and gene ontology (GO) terms for our gene list [30]. We selected the “Homo sapiens” species database and gene symbol as the gene identifier in DAVID and used the KEGG pathway as the background database. We visualized the enriched terms using a bar plot and performed gene set enrichment analysis using Excel 2019.

## 3. Results

Of the 3123 articles retrieved, 77 articles that met the inclusion criteria were included in this review. Out of them, seven were related to CVDs; five were related to T2D; six were related to obesity, and one was related to physical activity, as they were considered risk factors for CVDs and T2D; fourteen were associated with smoking and fifteen, with alcohol consumption; and others were related to drug addiction (three on cocaine, ten on heroin, five on opioids, three on amphetamine, and eight on substance abuse), as they can be risk factors for CVDs as well. Regarding the study designs, the majority of the studies were case–control (n = 50), seventeen were cross-sectional, seven were cohort, and three were randomized controlled trials.

Overall, 117,197 participants were included in 77 studies. Out of them, 27,883 were Asian (65.9% were Chinese), 39,727 were European (16% were European Americans), 6248 were African American, and 158 were Hispanic, although ethnicity was either reported as “Other” or not reported for 49,587 participants. A total of 174 SNVs in 69 different genes of the MCL system that were related to CVDs, T2D, and their potential risk factors were identified. Details on the identified genes and SNVs, including their IDs and other genomic features, are provided in Supplementary Appendix S2 and Supplementary Table S4. The findings were analyzed based on their themes (CVDs, T2D, obesity, smoking and nicotine dependence, alcohol dependence, drug addiction, and exercise behavior), which were related to the review question. Significant and non-significant SNVs for each gene are summarized under those thematic headings in Table 2. Notably, the significant SNVs associated with cardiovascular diseases were related to coronary artery disease, hypertension, venous thrombosis, atherosclerosis, and heart rate.

Our systematic review identified a significant association between the COMT gene and various themes related to CVDs, T2D, and their risk factors. The COMT gene was found to be significantly related to hypertension, dyslipidemia, insulin resistance, obesity, and drug abuse. The rs4680 SNP within the COMT gene was the most frequently reported genetic variant associated with these diseases and their risk factors. This SNP has been shown to affect the activity of the COMT enzyme, which may impact various physiological processes related to CVDs and T2D.

**Table 1:** Characteristics of the included articles (n = 77).

No.	First Author, Year	Country	Risk Factor/Disease	Sample Size (Male)	Study Design
1	Adamska-Patruno et al., 2019 [31]	Poland	Obesity	927 (473)	Case–control
2	Al-Eitan et al., 2012 [32]	Jordan	Drug use	460 (220)	Case–control
3	Aliasghari et al., 2021 [33]	Iran	Obesity	531 (0)	Case–control
4	Anney et al., 2007 [34]	Australia	Substance dependence	815 (–)	Cohort study
5	Aroche et al., 2020 [35]	Brazil	Crack cocaine addiction	1069 (605)	Case–control
6	Avsar et al., 2017 [36]	Turkey	Obesity	448 (142)	Case–control
7	Bach et al., 2015 [37]	Germany	Alcohol dependence	81 (43)	Cross-sectional
8	Batel et al., 2008 [38]	France	Alcohol dependence	230 (138)	Case–control
9	Beuten et al., 2006 [39]	USA	Nicotine dependence	2037 (668)	Cross-sectional
10	Beuten et al., 2007 [40]	USA	Nicotine dependence	2037 (–)	Cohort study
11	Céspedes et al., 2021 [41]	Brazil	Alcohol dependence	401 (366)	Case–control
12	Carr et al., 2014 [42]	USA	Obesity	245 (119)	Cross-sectional
13	Clarke et al., 2014 [43]	USA	Opioid and cocaine addiction	3311 (1554)	Case–control
14	da Silva Junior et al., 2020 [44]	Brazil	Alcohol dependence	300 (300)	Case–control
15	Doehring et al., 2009 [45]	Germany	Opioid dependence	88 (62)	Case–control
16	Erlich et al., 2010 [28]	USA	Nicotine and opioid dependence	505 (153)	Cross-sectional
17	Fedorenko et al., 2012 [46]	Russia	Alcohol dependence	501 (501)	Case–control
18	Fehr et al., 2013 [47]	Germany	Alcohol dependence	1159 (804)	Case–control
19	Fernàndez-Castillo et al., 2010 [48]	Spain	Cocaine dependence	338 (142)	Case–control
20	Fernàndez-Castillo et al., 2013 [49]	Spain	Cocaine dependence	914 (755)	Case–control
21	Flanagan et al., 2006 [50]	USA	Drug addiction (cocaine, alcohol, heroin, methadone, and methamphetamine)	1024 (–)	Case–control
22	Ge et al., 2015 [51]	China	Blood pressure and lipid level	3079 (1864)	Cohort study
23	Gellekink et al., 2007 [8]	Netherland	Venous thrombosis	607 (302)	Case–control
24	Gold et al., 2012 [52]	USA	Smoking cessation	1217 (553)	RCT
25	Hall et al., 2014 [53]	USA	CVD, aspirin and vitamin E	23,273 (0)	RCT
26	Hall et al., 2016 [54]	USA	T2D	909 (0)	Cross-sectional
27	Harrell et al., 2016 [55]	USA	Smoking	96 (71)	Cross-sectional
28	Huang et al., 2009 [56]	USA	Nicotine dependence	2037 (–)	Cohort study
29	Johnstone et al., 2004 [57]	USA	Smoking behavior	975 (399)	Cohort study
30	Joshua WB, 2013 [58]	USA	Obesity and drug abuse	59 (29)	Cross-sectional
31	Kaminskaite et al., 2021 [59]	Lithuania	Alcohol dependence	329 (127)	Case–control
32	Kishi et al., 2008 [7]	Japan	Meth use disorder	944 (479)	Case–control
33	Ko et al., 2012 [60]	China	Atherosclerosis	1503 (696)	Cross-sectional
34	Koijam et al., 2021 [61]	India	Heroin dependence	279 (110)	Case–control
35	Kring et al., 2009 [62]	Denmark	T2D and obesity	1557 (1557)	Cross-sectional
36	Kuo et al., 2018 [63]	China	Amphetamine dependence	1063 (854)	Case-control
37	Lachowicz et al., 2020 [64]	Poland	Polysubstance addiction	601 (601)	Case–control
38	Landgren et al., 2011 [33]	Sweden	Alcohol dependence	115 (88)	Case–control
39	Långberg et al., 2013 [65]	Sweden	Obesity and Type 2 diabetes	1177 (827)	Case–control
40	Levran et al., 2015 [66]	USA	Heroin (OD) and cocaine (CD) addictions	522 (281)	Case–control
41	Li et al., 2006 [67]	China	Heroin dependence	420 (–)	Cross-sectional
42	Li et al., 2016 [68]	China	Heroin addiction	1080 (–)	Case–control
43	Lind et al., 2009 [69]	Australia	Alcohol consumption behavior	305 (305)	Case–control
44	Lohoff et al., 2009 [70]	USA	Cocaine dependence	608 (328)	Case–control
45	Ma et al., 2005 [71]	USA	Nicotine dependence	2037 (686)	Case–control
46	Ma et al., 2018 [6]	China	Coronary artery disease	611 (471)	Case–control
48	Mattioni et al., 2022 [72]	France	Alcohol use, nicotine, and cannabis dependence	3056 (1834)	Case–control
47	Mir et al., 2018 [73]	India	Cardiovascular disease	200 (96)	Cohort study
49	Mutschler et al., 2013 [74]	Germany	Smoking behavior	551 (–)	Case–control
50	Najafabadi et al., 2005 [75]	Iran	Opium dependence	230 (230)	Case–control
51	Nelson et al., 2014 [76]	USA and Australia	Heroin dependence	3485 (2095)	Case–control
52	Noble et al., 1994 [77]	USA	Smoking	354 (190)	Case–control
53	Peng et al., 2013 [78]	China	Heroin dependence	844 (436)	Case–control
54	Perez de los Cobos et al., 2007 [79]	Spain	Heroin dependence	426 (305)	Case–control
55	Prado-Lima et al., 2004 [80]	Brazil	Smoking behaviors	625 (266)	Cross-sectional
56	Ragia et al., 2013 [81]	Greek	Smoking initiation	410 (215)	Case–control
57	Ragia et al., 2016 [82]	Turkey	Alcohol dependence	146 (111)	Case–control
58	Schacht et al., 2009 [9]	USA	Smoking marijuana	40 (30)	Cross-sectional
59	Schacht et al., 2022 [83]	USA	Alcohol dependence	87 (33)	RCT
60	Shiels et al., 2009 [84]	USA	Smoking	10,059 (3873)	Cross-sectional
61	Sipe, et al., 2002 [85]	USA	Drug users (drugs, alcohol, nicotine)	2881 (–)	Case–control
62	Spitta et al., 2022 [86]	Germany	Alcohol dependence	29 (26)	Case–control
63	Suchankova et al., 2015 [87]	USA	Alcohol dependence	2671 (2405)	Case–control
64	Sun et al., 2021 [88]	China	Methamphetamine, heroin, and alcohol addiction	6146 (4364)	Case–control
65	Tyndale et al., 2006 [89]	Canada	Drug addiction	749 (242)	Cross-sectional
66	Van Der Mee et al., 2018 [90]	Greece	Exercise behavior	12,929 (5144)	Cohort study
67	Vereczkei et al., 2013 [91]	Hungary	Heroin dependence	858 (597)	Case–control
68	Voisey et al., 2011 [92]	Australia	Alcohol, nicotine, and opiate dependence	748 (443)	Case–control
69	Wang et al., 2018 [93]	China	Coronary artery disease	707 (311)	Case–control
70	Wei et al., 2012 [94]	China	Nicotine dependence	480 (480)	Cross-sectional
71	Xie et al., 2013 [95]	China	Heroin addiction	533 (533)	Case–control
72	Xiu et al., 2015 [96]	China	Type 2 diabetes	1320 (758)	Case–control
73	Xu et al., 2004 [97]	Germany and China	Heroin dependence	1462 (–)	Case–control
74	Ying et al., 2009 [98]	China	Obesity	426 (217)	Case–control
75	Yu et al., 2006 [99]	USA	Nicotine dependence	1590 (730)	Cross-sectional
76	Zain et al., 2015 [100]	Pakistan	Type 2 diabetes	191 (107)	Cross-sectional
77	Zhu et al., 2013 [101]	China	Opioid dependence	939 (343 *)	Case–control
Total number of participants (accumulative)				117,197 (43,839)	

* = Number of males available for cases only, – = no data available on gender, RCT = randomized controlled trial.

**Table 2:** Single nucleotide polymorphisms encoding proteins of the MCL system that are related to cardiovascular diseases, type 2 diabetes, and their risk factors.

No.	Risk Factor/Disease	Gene Name ^‡^	Significant SNVs ^†^	Non-Significant SNVs ^†^
1	Cardiovascular diseases (CVDs)	*AP2A2*	rs7396366 [6]	
		*BZRAP1*		rs2526378 [93]
		*COMT*	rs4680 [51,53,60,73] Haplotype: rs2097603–rs4633–rs4680–rs174699 (G–C–G–T) [8] rs4633 [60] rs4818 [53]	(rs2097603 rs4633 rs174699) [8] Haplotypes: rs2097603–rs4633–rs4680–rs174699 (A–C-G–T, A–T-A–T, A–C–G–C) [8]
		*FAAH*	C385A (rs324420) [9]	
		*GLP1R*	rs4714210 [6]	(rs761387 rs2268635 rs7769547 rs910162 rs3765468 rs3765467 rs3765466 rs10305456 rs10305518 rs1820) [6]
2	Type 2 diabetes (T2D)	*5HT2A*		rs6311 [62]
		*5HT2C*	rs3813929 [62]	
		*ADRA2A*	(rs553668 rs521674) [65]	rs11195419 [65]
		*COMT*	rs4646312 [96] rs4680 [54,62,96] (900 I/D C) [100] (rs4633 rs4818) [54]	
		*DRD3*		(rs167771 rs324029 rs8076005 rs20667) [96]
		*SLC6A4*	Haplotypes: rs4646312, rs4680 (C–G, T–A) [96] Diplotype: rs4646312–rs4680 (C–G_T–G) SNP–SNP interactions Additive × additive (rs4680 × rs2066713) Dominant × dominant (rs4680 × rs2066713) [11]	Haplotypes: rs8076005, rs2066713 (A–A, A–G, G–G) [96]
3	Obesity	*5HT2AR*	–c.1438 A>G [98]	
		*5HT2C*	Combined genotype with *COMT* (rs3813929 rs4680) [62]	
		*ANNK1*	rs1800497 [33]	
		*ADRA2A*	(rs553668 rs521674) [65]	rs11195419 [65]
		*COMT*	rs4680 [62]	rs4580 [42]
		*DAT1*		rs28363170 [42]
		*DBH*		(rs77905 rs6271 rs1611115 rs1108580) [42]
		*DDC*		(rs2060762 rs11575543 rs11575542 rs11575522 rs11238131) [42]
		*DRD1*		rs4532 [42]
		*DRD2*	rs1799732 [33]	rs1800497 [42] (rs1800498 rs6277) [72]
		*DRD3*		rs6280 [42]
		*DRD4*		rs4646984 [42]
		*HTR1A*		(rs6295 rs1800044 rs1799920 rs10042486) [42]
		*HTR1B*		(rs6296 rs13212041 rs130058) [42]
		*HTR2A*	rs6314 [42]	(rs927544 rs7997012 rs6313 rs6311 rs2770296 rs1923886) [42]
		*LEPR*	rs1137100 [58]	rs1137101 [58]
		*MAOA*	MAOA-LPR (3.5R/4R) [42] u VNTR [36]	
		*MC4R*	(rs1350341 rs17782313 rs633265) [31]	
		*OPRD*		(rs569356 rs2236861 rs204076 rs7773995 rs514980 rs2281617 rs1799971 rs12205732 rs10485057 rs17174801) [42]
		*SERT*		(rs2066713 rs2020933 rs16965628 rs1042173) [42]
		*SPR*		(rs2421095 rs1876487) [42]
		*TH*		rs71029110 [42]
		*TPH2*		(rs7963720 rs7305115 rs4290270 rs17110690 rs1487275 rs17110747) [42]
4	Smoking and nicotine dependence	*5HT2A*	T102C [80]	
		*ANKK1*	(rs11604671 rs2734849) [56]	(rs10891545 rs7945132 rs4938013 rs7118900 rs1800497) [56]
		*CHRNA3*	(rs660652 rs1051730) [28]	(rs6495308 rs12443170) [28]
		*CHRNA4*	rs2236196 [94]	
		*CHRNA5*	(DRD2/5-HT2CR –759C>T genotype combinations: A1–/–759T–, A1+/–759T–, A1–/–759T + A1+/–759T+; DRD2/5-HT2CR –697G>C genotype combinations: A1–/–697C–, A1+/–697C–, A1–/–697C+ A1+/–697C+, 5-HT2CR –759C>T; interaction of 5-HT2CR –759C>T and DRD2 TaqIA; 5-HT2CR –697G>C; interaction of 5-HT2CR –697G>C and DRD2 TaqIA) [28] (rs936460 rs936461 rs12280580) [55]	rs16969968 [28]
		*CHRNB3*	rs4954 [94] rs660652 [28]	
		*COMT*	rs4680 [39,84] (rs740603 rs4680 rs174699 rs933271 rs174699) [39] Haplotype: rs740603–rs4680–rs174699 (A–G–T) rs933271–rs4680–rs174699 (T–G–T, C–A–T) [39]	rs4633 [39] rs4680 [74]
		*DBH*	rs77905 [84]	
		*DDC*	rs11575461 [94] (rs12718541 rs1470747 rs11238214 rs2060761) [99] rs921451 [71,99] Haplotype: rs921451–rs3735273–rs1451371–rs2060762 (T–G–T–G) rs921451–rs3735273–rs1451371–rs3757472 (T–G–T–G) [71]	(rs11575542 rs732215 rs1451371 rs3823674 rs1470750 rs11575334 rs4947644) [99] (rs998850 rs3735273 rs1470750 rs1451371 rs732215 rs3757472 rs2060762) [71]
		*DRD2*	(rs11214613 rs6589377) [94] TaqIA1 [77]	(rs6278 rs6279 rs1079594 rs6275 rs2075654 rs2587548 rs2075652 rs1079596 rs4586205 rs7125415 rs4648318 rs4274224 rs7131056 rs4648317 rs4350392 rs6589377) [56] C32806T [57] (rs1800498 rs6277) [72]
		*DRD3*	rs2630351 [94]	
		*DRD4*	(rs936460 rs936461 rs12280580) [55]	rs1805186 [55]
		*DRD5*	rs1967550 [94]	
		*FIGNL1*	rs10230343 [99]	
		*GABBR2*	rs2779562 [40]	
		*GALR1*	rs2717162 [52]	
		*GRB10*		(rs12669770 rs12540874 rs2715129) [99]
		*MAOA*	rs1801291 [84]	
		*MAP3K4*	rs2314378 [94]	
		*PPP1R1B*	Haplotype: rs2271309–rs907094–rs3764352–rs3817160 (–C–T–G–C) rs879606 [40]	rs1874228 [40]
		*ZNFN1A1*		(rs11980407 rs1110701) [99]
5	Alcohol dependence	*ADH1B*	rs1229984 [88]	
		*AGBL4*		rs147247472 [88]
		*ANKK1*		rs1800497 [59] (rs4938015 rs1800497) [72,86]
		*ANKS1B*		rs2133896 [88]
		*CHRNA3*		(rs6495307 rs1317286 rs12443170 rs8042059) [34]
		*CHRNA4*		(rs1044396 snp12284 rs6011776 rs6010918) [34]
		*CHRNA6*		(rs17621710 rs10087172 rs10109429 rs2196129 rs16891604) [34]
		*CHRNB2*		(rs2072659 rs2072660) [34]
		*CHRNB3*	rs13261190 [34]	(rs62518216 rs62518217 rs62518218 rs16891561) [34]
		*COMT*	(rs165774 rs4680) [59,83] Haplotype: rs4680–rs165774 (–A–A) [92]	(rs4633 rs740602 rs4818 rs4680 rs4646315) [41]
		*CRH*	rs6999100 [58]	
		*CSNK1E*	rs135745 [58]	
		*CTNNA2*		rs10196867 [88]
		*DDC*	rs11575457 [41]	(rs5884156 rs4490786 rs11575457 rs58085392 rs2876829 rs11575375 rs3735273 rs6950777 rs6264) [41]
		*DAT1*	(rs6350 rs463379) [69]	(rs10064219 rs12516948 rs40184 rs6347 rs464049 rs403636) [69]
		*DRD1*	rs686 [38] (rs2283265 rs1076560 rs2075654 rs1125394 rs2734836 rs1799732) [32] Haplotype: rs686–rs4532 (–T–G) [38]	(rs686 rs155417 rs4532) [41]
		*DRD2*	(rs6277 rs1800498) [72]	A2/A1 [82] rs1800497 [34] (rs6277 rs6275 rs1076560 rs35352421 rs11608185 rs12808482) [41]
		*DRD3*		Ser9Gly [82] (rs149281192 rs2251177 rs3732783 rs6280) [41]
		*DRD4*		rs7124601
		*DRD5*		(rs2076907 rs6283 rs1967551) [41]
		*DβH*		1021 C/T [82]
		*FAAH*	385 C/A [85]	
		*GHRL*	(rs42451 rs35680) [34]	(rs4684677 rs34911341 rs696217 rs26802) [34]
		*GHSR*	rs495225 [34]	(rs2948694 rs572169 rs2232165) [34]
		*GLP1R*	(rs7766663 rs2235868 rs7769547 rs10305512 rs2143734 rs2268650 rs874900 rs6923761 rs7341356 rs932443 rs2300613) [87]	(rs7738586 rs9296274 rs2268657 rs3799707 rs3799707 rs910170 rs1042044 rs12204668 rs1076733 rs2268640 rs2206942 rs10305514 rs4714210 rs4254984 rs9968886) [87]
		*GRIK1*		rs2832407 [82]
		*HTR2A*	(rs6313 rs6311) [44]	
		*OPRM1*	rs1799971 [37]	A118G [82]
		*PIP4K2A*	(rs746203 rs2230469) [46]	(rs8341 rs943190 rs1132816 rs1417374 rs11013052) [46]
		*SLC6A3*		(rs429699 rs8179029 rs6347 rs6348 rs460000 rs465130 rs465989 rs13189021 rs2254408 rs2270914 rs2270913 rs8179023 rs6350) [41]
		*TH*		(rs6578990 rs12419447 rs6357 rs7925924 rs4074905 rs6356 rs7925375) [41]
		*VMAT2*	rs363387 [47] Haplotypes: rs363332, rs363387 (–G–T, –G–G) rs363387–rs363333 (–T–T) rs363333–rs363334 (C–T) rs363387–rs363333–rs363334 (–T–T–C) rs363332–rs363387–rs363333–rs363334 (–G–T–T–C) [47]	(rs363371 rs363324 rs11197931) [47]
6	Drug addiction	*ADH1B*		rs1229984 [88]
		*AGBL4*	rs147247472 [88]	
		*ANKK1*	(rs877137 rs877138 rs12360992 rs4938013 rs2734849 rs2734848) [76] rs1800497 [45,91]	rs1800497 [76] rs7118900 [66]
		*ANKS1B*	rs2133896 [88]	
		*CDNF*		(rs11259365 rs7094179 rs7900873 rs2278871) [70]
		*CHRM5*	rs7162140 [102]	(rs661968 257A>T rs2702309 rs2702304 rs2576302 rs2705353) [102]
		*CHRNA4*		(rs755203 rs2273506 rs2273505 rs3787141 rs3787140 rs2273504 rs2273502 rs2273501 rs1044396 rs1044397 rs3787137 rs2236196 rs4522666) [7]
		*CHRNA5*	rs16969968 [35] Haplotypes: rs16969968–rs660652–rs1051730–rs6495308–rs12443170 (A–G–A–T–G, G–G–G–T–G)) [28] (rs588765 rs514743) [35]	
		*CHRNB2*		(rs4845652 rs2072658 rs2072659 rs2072660 rs3811450) [7]
		*CNTFR*	rs7036351 [49]	
		*COMT*	rs4680 [66]	rs4680 [91] (rs933271 rs2239393 rs4818) [66] (rs265981 rs1800497 VNTR 130–166 bp rs2519152 VNTR) [90]
		*CSNK1E*		rs5757037 [66]
		*CTNNA2*	rs10196867 [88]	
		*DAT1*	Int8 VNTR [48] (rs28363170 rs3836790 rs246997) [61]	SLC6A3 VNTR [67] 3′UTR VNTR [48] (rs40184 rs27048 rs37021 rs250683 rs250682 rs427284) rs458609) [61]
		*DBH*	rs6479643 [49]	rs1611115 [95] rs1108580 [66] 1021C>T [81] (rs1108580 5UTR ins/del) [48] rs2519152 [90]
		*DCC*	(rs16956878 rs12607853 rs2292043) [68]	(rs2122822 rs2329341) [66] (rs17753970 rs934345 rs2229080) [68]
		*DLG2*		(rs575050, rs2512676, rs17145219, rs2507850) [68]
		*DRD1*	(rs4532 rs686) [101]	(rs4532 rs5326 rs2168631 rs6882300 rs267418) [78] (rs686 rs5326) [66] (rs10078866 rs10063995 rs5326 rs1799914 rs4867798) [101] rs265981 [90]
		*DRD2*	TaqI A1 [67,75,79] (rs2234689 rs1554929 rs2440390 rs1076563) [76] rs1079597 [91] rs1076560 [43,45] (241 A>G; TaqIB A>G; TaqID G>A; and intron 4 T>C) [97] (759 C>T; 697 G>C) [81] Haplotypes: rs1076560, rs1800498, rs1079597, rs6276, and rs180049 of the ANKK1 (C–T–G–A–T, C–T–G–A–C) [64]	rs7125415 [76] (141 ins/del C; intron 6 ins/del G; 311 Ser>Cys; 20236 C>T; exon 822640 C>G; and TaqIA G>A) [97] rs1800498 [72,91] (rs1076560 rs2283265 rs2587548 rs1076563 rs1079596 rs1125394 rs2471857 rs4648318 rs4274224 rs1799978) [66] TaqIA [81] rs1079597 [48] rs1800497 [48,90] (rs12364283 rs1799978 rs1799732 rs4648317 rs1800496 rs1801028 rs6275 rs6277) [45,72]
		*DRD3*	Haplotype: rs324029–rs6280–rs9825563 (A–T–A) rs2134655–rs963468–rs9880168 (A–T–A) [63]	(rs3773678 rs167771) [66] rs6280 [90] (rs2046496 rs2630351) [63]
		*DRD4*	rs1800955 [91]	(rs936462 rs747302) [91] VNTR 48 bp [90]
		*DRD5*		DRP (A9/A9) [67] rs2867383 [66] VNTR 130–166 bp [90]
		*FAAH*	(rs12075550 rs6658556 796A>G rs932816 rs4660930) [50]	385 C/A * [50,89]
		*FAT3*		(rs10765565 rs4753069 rs2197678 rs7927604) [68]
		*HTR1E*	rs1408449 [49]	
		*HTR2A*	(rs6561332 rs6561333) [49]	
		*KTN1*		(rs10146870 rs1138345 rs10483647 rs1951890 rs17128657 rs945270) [68]
		*NCAM1*	(rs4492854 rs587761) [76]	rs11214546 [76]
		*NGFR*	rs534561 [49]	
		*NTF3*	rs4073543 [49]	
		*NTRK2*	rs1147193 [49]	
		*NTRK3*	(rs12595249 rs744994 rs998636) [49]	
		*TH*	rs2070762 [49]	
		*TTC12*	(rs2303380 rs10891536 rs4938009 rs7130431 rs12804573) [76]	rs719804 [76]
7	Exercise Behavior	*COMT*	rs4680 [90]	
		*DAT1*	VNTR 440 bp [90]	
		*DBH*		rs2519152 [90]
		*DRD1*		rs265981 [90]
		*DRD2*/*ANKK1*		rs1800497 [90]
		*DRD3*		rs6280 [90]
		*DRD4*	VNTR 48 bp (7r) [90]	
		*DRD5*		VNTR 130–166 bp [90]
		*MAOA*		VNTR 30 bp [90]

^‡^ A concise summary of the role of each gene and the chromosome where it is located is provided in Supplementary Table S4, ^†^ “Significant” denotes SNVs with a statistically significant association with CVDs, T2D, and/or their risk factors, while “Non-Significant” indicates SNVs without a statistically significant association, * significant with regular sedative users only.

**Figure 2:**
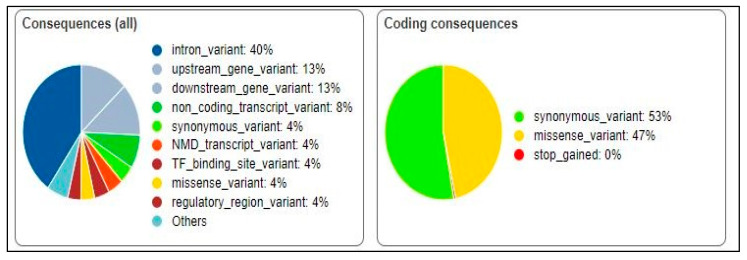
Predicted effects of genetic variants on protein function.

The significant SNVs were analyzed using the VEP tool [29]. The predicted effects of the genetic variants on protein function were synonymous (53%) and missense (47%) (Figure 2). Further analysis of the missense variants using VEP revealed that 48.2% were predicted to be benign, 3.38% were predicted to be likely benign, and 18.42% were predicted to initiate a drug response.

Moreover, cellular component and functional enrichment analyses of the 69 identified genes were performed using DAVID [30]. For the cellular component enrichment analysis, we found that genes were significantly enriched in several cellular components, including serotonergic and dopaminergic synapses. These results suggest that the 69 genes are involved in various cellular processes and may play important roles in CVDs and T2D development. We also performed a functional enrichment analysis. We found that the 69 genes were significantly enriched in several functional pathways, including “dopamine neurotransmitter receptor activity”, “dopamine binding”, and “serotonin binding”. These pathways are known to be involved in various aspects of CVD and T2D development and progression. The top ten terms for the cellular components, functional enrichments, and phenotypic enrichments of the identified genes are provided in Supplementary Figures S1–S3.

## 4. Discussion

The MCL system, originating in the VTA region of the brain, is known to affect a person’s adverse health behaviors, which increase their risk for CVDs and T2D development [103,104]. Overstimulation of dopamine, as the main neurotransmitter of the MCL, will lead to craving for different substances, and thus, might be related to increasing the risk of developing CVDs and T2D [9]. Numerous genes in the MCL system have been found to be related to CVDs and T2D, either directly or indirectly, through their involvement in different risky behaviors [8,51,53,54,60,62,73,96]. MCL genes that were frequently found to be associated with multiple traits are discussed herein.

The catechol-O-methyltransferase (*COMT*) gene was found to be significantly related to all themes of this study. The *COMT* enzyme is encoded by the *COMT* gene, as it is responsible for the degradation of dopamine–adrenaline and noradrenaline, and catecholamine [73]. Studies show that regulating dopamine activities might have an impact on vascular resistance [73] and numerous reward behaviors like obesity [62]. The rs4680 (*Val158Met*) of the *COMT* gene was the most prevalent SNV that was related not only to CVDs [8,51,53,60,73] but also to T2D [54,62,96] and other risk factors [22,39,62,68,76,105]. A case–control study among subjects of European ancestry found no significant association between rs4680 and nicotine dependence when using the Fagerstrom Test for Nicotine Dependence (FTND) [74]. However, the same measurement tool revealed a significant association among two ethnic groups (African American and European American) [39]. Furthermore, a study showed a positive relationship between rs4680 and smoking initiation among females and with smoking persistence among males, as smoking status was self-reported, but not with other smoking behaviors. This variation might be due to the absence of a standard measurement tool for smoking behaviors [39].

In regards to drug addiction and rs4680, two case–control studies [66,91] have shown contradictory results for heroin addiction, even though the same standard instrument (Diagnostic and Statistical Manual of Mental Disorders, 4th edition) was applied for both. A study revealed that African American descent were genetically susceptible to heroin addiction, as the *Val* allele of the *COMT* gene is a risk allele [66]; in contrast, no relationship was found in another study conducted among people of European descent only [91]. These reversing findings might be attributed to the diversity in the ethnic groups and sample sizes of the studies.

A release of mesocorticolimbic dopamine is modulated by a CB1 receptor that is inactivated by fatty acid amide hydrolase (*FAAH*) enzymes, triggering different aspects of addiction [9,50,89]. An SNV variant (rs324420/C385A) of the *FAAH* gene was found to establish important risk factors for alcohol dependence [50] and marijuana use [9]. Under the recessive model of C385A, it was found to be related to increased heart rate following cannabis smoking [50]. This proved the connection between MCL and drug addiction, which is considered a risk factor for CVDs. However, a study with a larger sample size conducted among adult Caucasians found that a variant of FAAH was not significantly associated with cannabis use [89]. Despite using the same diagnostic criteria for substance use disorder (DSM-IV) in the studies by Schacht et al. [9] and Flanagan et al. [50], the heterogeneity of the sample size, ethnicity, and inclusion criteria might have contributed to the variety in the correlation between the *FAAH* variant and substance use.

The glucagon-like peptide-1 *(GLP-1*) is a hormone that regulates appetite and food intake [6,87], and its receptor activation might affect the reduction in driven behavior for alcohol use [87,106]. *GLP-1R* in the mesolimbic area is involved in food-related reward processing [6,87]. *GLP-1R* agonists have a consequence on CVDs through their physiological effects like reduction in fatty acid absorption, increased satiety, and reduction in body weight [6,87]. The risk of coronary artery diseases (CADs) was found to be lower among individuals who carried the GG genotypes of the rs4714210 variant of the *GLP-1R* gene than for AA genotype carriers [107]; however, another study that addressed the targeted SNVs of *GLP-1R* for the treatment of alcohol use disorder (AUD) among Caucasians and African Americans indicated no relationship between rs4714210 and AUD [106]. On the other hand, rs7769547 of the *GLP-1R* gene was significantly associated with AUD [87], but not with that of CADs [6]. This might be due to the fact that different phenotypes were considered; as a consequence, one variant might be a risk for a particular phenotype but not for others.

Different substances such as nicotine, cocaine, alcohol, opiates, and food increase brain dopamine levels and activate the MCL dopaminergic reward pathways of the brain, hence resulting in various risky behaviors such as smoking, alcohol dependence, and obesity [42,67,75,77,79,82,94]. There are five dopamine receptor genes, *DRD1, DRD2*, *DRD3, DRD4*, and *DRD5*, which are mainly related to different risky behaviors like substance abuse and addiction [32,38,42,55,63,67,75,77,79,90,94,101]. They are considered risk factors for CVDs and T2D. *DRD2 TaqI A* is an SNV with two variants: A1, the less frequent allele, and A2, the most frequent. The A1 allele is related to a reduction in the concentrations of D2 receptors which results in diverse substance use disorders (SUDs). Studies have identified that *TaqI A* is significantly associated with smoking [77], heroin [67,79], and opium addiction [75]. On the other hand, Ragia et al. [81] showed no interaction between the *DRD2 TaqI A* polymorphism and smoking initiation; however, they indicated that an interaction between *DRD2 TaqI A1* and *5-HT2CR -759T* alleles resulted in smoking initiation behavior [81].

Though the genetic risk factors for CVDs and T2D are abundant, no fundamental study has yet been conducted to study all MCL genetic variants in a comprehensive manner. Intensively studying the impacts of these SNVs on chronic diseases might pave the way for establishing new preventive and treatment approaches. Therefore, this systematic review was conducted to compile worthwhile SNVs encoding proteins of the MCL system that were associated with CVDs and T2D. Although some published studies did not consider ethnicity and gender as cofounders, the available data from the literature seem to designate that the MCL system has a strong relationship with increasing the risk of developing CVDs and T2D, either directly or indirectly through modifying their risk factors. Dimorphisms in gender and ethnicity among the included studies might have contributed to the heterogeneity of the outcomes of this review. Another limitation would be that relying on aggregated data restricted our ability to analyze individual patient data, curtailing detailed insights into specific subpopulations. While our comprehensive search strategy aimed to minimize bias in study selection, it is imperative to acknowledge the underrepresentation of studies in languages other than English. Moreover, interpreting biological causality remains challenging; although our review identified statistically significant associations, establishing causation necessitates a more nuanced understanding of the underlying biological mechanisms. Future research should rigorously explore molecular pathways to enhance comprehension. The generalizability of our findings is inherently constrained by the variations in the included study populations, methodologies, and geographic locations, thereby limiting the external validity of our results. Altogether, further studies using these SNVs might help in developing a better understanding of how these SNVs alter CVDs and T2D.

## Data Availability

Not applicable.

## Supplementary Materials

The following supporting information can be downloaded at: https://www.mdpi.com/article/10.3390/genes15010109/s1, Figure S1: The top ten cellular component enrichment terms of the identified genes; Figure S2: The top ten functional enrichment terms of the identified genes; Figure S3: The top ten phenotypic enrichment terms of the identified genes; Table S1: Keywords used for PubMed search performed on 2023-03-06; Table S2: Search strategy on PubMed; Table S3: Search strategy on Web of Science; Table S4: Gene Catalog: Chromosome Assignment and Functional Roles.
