# Supplementary material for: Single Nucleotide Variants (SNVs) of the Mesocorticolimbic System Associated with Cardiovascular Diseases and Type 2 Diabetes: A Systematic Review
Source: Genes (Basel). 2024 Jan 17;15(1):109. doi: 10.3390/genes15010109 (PMC10815084; doi:10.3390/genes15010109)
Supplement: Supplementary file 1 [file genes-15-00109-s001.zip › Suppl_files 2024-01-02.pdf]

Supplementary Materials

Single Nucleotide Variants (SNVs) of the Mesocorticolimbic System Associated with Cardiovascular Diseases and Type 2 Diabetes: A Systematic Review

Mohammed Abdulridha Merzah [1,2], Shewaye Fituma Natae [1,2], János Sándor [1,3] and Szilvia Fiatal [1*]

[1]Department of Public Health and Epidemiology, Faculty of Medicine, University of Debrecen, 4032 Debrecen, Hungary
[2]Doctoral School of Health Sciences, University of Debrecen, 4032, Debrecen, Hungary
[3]ELKH-DE Public Health Research Group, Department of Public Health and Epidemiology, Faculty of Medicine, University of Debrecen, 4032 Debrecen, Hungary

List of Tables:

Table S1: Keywords used for PubMed search performed on 2023-03-06 2
Table S2: Search strategy on PubMed 3
Table S3: Search strategy on Web of Science 7
Table S4: Gene Catalog: Chromosome Assignment and Functional Roles 8

List of Figures:

Figure S1: The top ten cellular component enrichment terms of the identified genes 10
Figure S2: The top ten functional enrichment terms of the identified genes 10
Figure S3: The top ten phenotypic enrichment terms of the identified genes 11

Supplementary Materials

Table S1: Keywords used for PubMed search performed on 2023-03-06

**BLOCKS**	
**1**	**Mesocorticolimbic**<br>1. Mesocorticolimbic (MeSH)<br>2. Ventral Tegmental Area (MeSH)<br>3. Dopaminergic pathway (MeSH)<br>4. Reward pathway (MeSH)<br><br>Filter- Humans, English, 2019:2022 (Publishing date)
**2**	**Cardiovascular**<br>1. Cardiovascular disease (MeSH)<br>2. CVDs / CVD(keyword)<br>3. Heart disease (keyword)
**3**	**Genetic variants**<br>1. Single nucleotide polymorph (MeSH)<br>2. Genetic susceptibility (MeSH)<br>3. Gene (keyword)<br>4. SNPs/SNP (keyword)<br>5. Allele (keyword)<br>6. Gene variant (keyword)
**4**	**Risk factors**<br>1. Risk behaviours (MeSH)<br>2. Unhealthy eating habits (MeSH)<br>3. Smoking (keyword)<br>4. Physical inactive(MeSH)<br>5. Alcohol (MeSH)<br>**6.** Substance abuse (MeSH)

** The search was updated on 31st of May 2023 using the same terms.

Supplementary Materials

Table S2: Search strategy on PubMed

Search	Query	Search details	Number of hits
**Mesocorticolimbic system**			
#1	Mesocorticolimbic	"mesocorticolimbic"[All Fields] OR "mesolimbic"[All Fields] OR "corticolimbic"[All Fields]	8,166
#2	Ventral Tegmental Area	"ventral tegmental area"[MeSH Terms] OR ("ventral"[All Fields] AND "tegmental"[All Fields] AND "area"[All Fields]) OR "ventral tegmental area"[All Fields]	10,040
#3	Dopaminergic pathway	("dopamine agonists"[Pharmacological Action] OR "dopamine agonists"[MeSH Terms] OR ("dopamine"[All Fields] AND "agonists"[All Fields]) OR "dopamine agonists"[All Fields] OR "dopaminergics"[All Fields] OR "dopamine"[MeSH Terms] OR "dopamine"[All Fields] OR "dopaminergic"[All Fields] OR "dopaminergically"[All Fields]) AND ("pathway"[All Fields] OR "pathway s"[All Fields] OR "pathways"[All Fields])	26,650
#4	Reward pathway	("reward"[MeSH Terms] OR "reward"[All Fields] OR "rewarding"[All Fields] OR "rewards"[All Fields] OR "reward s"[All Fields] OR "rewarded"[All Fields]) AND ("pathway"[All Fields] OR "pathway s"[All Fields] OR "pathways"[All Fields])	6,382
#5	#1, #2, #3, & #4	(("reward"[MeSH Terms] OR "reward"[All Fields] OR "rewarding"[All Fields] OR "rewards"[All Fields] OR "reward s"[All Fields] OR "rewarded"[All Fields]) AND ("pathway"[All Fields] OR "pathway s"[All Fields] OR "pathways"[All Fields])) OR (("dopamine agonists"[Pharmacological Action] OR "dopamine agonists"[MeSH Terms] OR ("dopamine"[All Fields] AND "agonists"[All Fields]) OR "dopamine agonists"[All Fields] OR "dopaminergics"[All Fields] OR "dopamine"[MeSH Terms] OR "dopamine"[All Fields] OR "dopaminergic"[All Fields] OR "dopaminergically"[All Fields]) AND ("pathway"[All Fields] OR "pathway s"[All Fields] OR "pathways"[All Fields])) OR ("ventral tegmental area"[MeSH Terms] OR ("ventral"[All Fields] AND "tegmental"[All Fields] AND "area"[All Fields]) OR "ventral tegmental area"[All Fields]) OR ("mesocorticolimbic"[All Fields] OR "mesolimbic"[All Fields] OR "corticolimbic"[All Fields])	41,332
#6	Filter #5 for non-used terms, English, and human only	("reward pathway*"[All Fields] OR "dopamine agonists"[All Fields] OR "dopaminergics"[All Fields] OR "dopaminergic"[All Fields] OR "dopaminergically"[All Fields] OR "ventral tegmental area"[All Fields] OR "mesocorticolimbic"[All Fields] OR "mesolimbic"[All Fields] OR "corticolimbic"[All Fields]) AND ((humans[Filter]) AND (english[Filter]))	**43,950**
**Cardiovascular Diseases**			
#7	Cardiovascular diseases	"cardiovascular diseases"[MeSH Terms] OR ("cardiovascular"[All Fields] AND "diseases"[All Fields]) OR "cardiovascular diseases"[All Fields]	2,785,129
#8	Cardiovascular disease OR CVDs OR CVD OR heart disease	"cardiovascular diseases"[MeSH Terms] OR ("cardiovascular"[All Fields] AND "diseases"[All Fields]) OR "cardiovascular diseases"[All Fields] OR ("cardiovascular"[All Fields] AND "disease"[All Fields]) OR "cardiovascular disease"[All Fields] OR "CVDs"[All Fields] OR "CVD"[All Fields] OR ("heart	3,037,186

		diseases"[MeSH Terms] OR ("heart"[All Fields] AND "diseases"[All Fields]) OR "heart diseases"[All Fields] OR ("heart"[All Fields] AND "disease"[All Fields]) OR "heart disease"[All Fields])	
#9	Filter #7 for non-used terms, English, and human only	("cardiovascular diseases"[All Fields] OR "CVDs"[All Fields] OR "CVD"[All Fields] OR "heart diseases"[All Fields]) AND ((humans[Filter]) AND (english[Filter]))	**260,233**
**Diabetes**			
#10	Diabetes Mellitus	"diabetes mellitus"[MeSH Terms] OR ("diabetes"[All Fields] AND "mellitus"[All Fields]) OR "diabetes mellitus"[All Fields]	589,095
#11	Diabetes mellitus OR diabetic OR diabetes	"diabetes mellitus"[MeSH Terms] OR ("diabetes"[All Fields] AND "mellitus"[All Fields]) OR "diabetes mellitus"[All Fields] OR ("diabete"[All Fields] OR "diabetes mellitus"[MeSH Terms] OR ("diabetes"[All Fields] AND "mellitus"[All Fields]) OR "diabetes mellitus"[All Fields] OR "diabetes"[All Fields] OR "diabetes insipidus"[MeSH Terms] OR ("diabetes"[All Fields] AND "insipidus"[All Fields]) OR "diabetes insipidus"[All Fields] OR "diabetic"[All Fields] OR "diabetics"[All Fields] OR "diabets"[All Fields]) OR ("diabete"[All Fields] OR "diabetes mellitus"[MeSH Terms] OR ("diabetes"[All Fields] AND "mellitus"[All Fields]) OR "diabetes mellitus"[All Fields] OR "diabetes"[All Fields] OR "diabetes insipidus"[MeSH Terms] OR ("diabetes"[All Fields] AND "insipidus"[All Fields]) OR "diabetes insipidus"[All Fields] OR "diabetic"[All Fields] OR "diabetics"[All Fields] OR "diabets"[All Fields])	918,179
#12	diabetes mellitus OR Diabet* OR insulin resistance OR metabolic disorder	"diabetes mellitus"[MeSH Terms] OR ("diabetes"[All Fields] AND "mellitus"[All Fields]) OR "diabetes mellitus"[All Fields] OR "diabet*"[All Fields] OR ("insulin resistance"[MeSH Terms] OR ("insulin"[All Fields] AND "resistance"[All Fields]) OR "insulin resistance"[All Fields]) OR ("metabolic diseases"[MeSH Terms] OR ("metabolic"[All Fields] AND "diseases"[All Fields]) OR "metabolic diseases"[All Fields] OR ("metabolic"[All Fields] AND "disorder"[All Fields]) OR "metabolic disorder"[All Fields])	**1,717,333**
#13	Filter #12 for non-used terms, English, and human only	("diabetes mellitus"[All Fields] OR "diabetes"[All Fields] OR "diabet*"[All Fields] OR "insulin resistance"[All Fields]) AND ((humans[Filter]) AND (english[Filter]))	**642,557**
Risk factors or unhealthy behaviour related to diabetes and CVDS			
#14	Risk behaviours	"risk behaviours"[All Fields] OR "risk taking"[MeSH Terms] OR "risk taking"[All Fields] OR ("risk"[All Fields] AND "behaviors"[All Fields]) OR "risk behaviors"[All Fields]	90,654
#15	Risk behav* OR unhealthy eating habits OR smok* OR physical inactiv* OR exercis* OR Alcohol OR substance abuse	(("risk"[MeSH Terms] OR "risk"[All Fields]) AND "behav*"[All Fields]) OR (("unhealthier"[All Fields] OR "unhealthiness"[All Fields] OR "unhealthy"[All Fields]) AND ("feeding behavior"[MeSH Terms] OR ("feeding"[All Fields] AND "behavior"[All Fields]) OR "feeding behavior"[All Fields] OR ("eating"[All Fields] AND "habits"[All Fields]) OR "eating habits"[All Fields])) OR "smok*"[All Fields] OR (("physical examination"[MeSH Terms] OR ("physical"[All Fields] AND "examination"[All Fields]) OR "physical examination"[All Fields] OR "physical"[All Fields] OR "physically"[All Fields] OR "physicals"[All Fields]) AND "inactiv*"[All Fields]) OR "exercis*"[All Fields] OR ("alcohol s"[All Fields] OR "alcoholate"[All Fields] OR "alcoholates"[All Fields] OR "alcoholic s"[All Fields] OR	2,340,445

Supplementary Materials

		"alcoholics"[MeSH Terms] OR "alcoholics"[All Fields] OR "alcoholic"[All Fields] OR "alcoholism"[MeSH Terms] OR "alcoholism"[All Fields] OR "alcoholisms"[All Fields] OR "alcoholism s"[All Fields] OR "alcoholization"[All Fields] OR "alcohols"[MeSH Terms] OR "alcohols"[All Fields] OR "ethanol"[MeSH Terms] OR "ethanol"[All Fields] OR "alcohol"[All Fields]) OR ("substance related disorders"[MeSH Terms] OR ("substance related"[All Fields] AND "disorders"[All Fields]) OR "substance related disorders"[All Fields] OR ("substance"[All Fields] AND "abuse"[All Fields]) OR "substance abuse"[All Fields])	
#16	Filter #15 for non-used terms, English, and human only	(("risk behav*"[All Fields] OR "unhealthy behav*"[All Fields] OR "eating habits"[All Fields] OR "smok*"[All Fields] OR "physical inactiv*"[All Fields] OR "alcohol*"[All Fields] OR "substance related disorders"[All Fields] OR "substance abuse"[All Fields]) AND ("humans"[MeSH Terms] AND "english"[Language])) AND ((humans[Filter]) AND (english[Filter]))	**641,798**
**Genetic variation**			
#17	Gene* OR SNPs OR SNP OR single nucleotide polymorph*	"gene*"[All Fields] OR ("polymorphism, single nucleotide"[MeSH Terms] OR ("polymorphism"[All Fields] AND "single"[All Fields] AND "nucleotide"[All Fields]) OR "single nucleotide polymorphism"[All Fields] OR "snps"[All Fields]) OR ("socioaffect neurosci psychol"[Journal] OR "snp"[All Fields]) OR (("single person"[MeSH Terms] OR ("single"[All Fields] AND "person"[All Fields]) OR "single person"[All Fields] OR "single"[All Fields] OR "singles"[All Fields]) AND ("nucleotid"[All Fields] OR "nucleotides"[MeSH Terms] OR "nucleotides"[All Fields] OR "nucleotide"[All Fields] OR "nucleotidic"[All Fields]) AND "polymorph*"[All Fields])	852,112
#18	Gene* OR SNPs OR SNP OR single nucleotide polymorph* OR morphism OR allele* OR gene variant OR genetic susceptibility	"gene*"[All Fields] OR ("polymorphism, single nucleotide"[MeSH Terms] OR ("polymorphism"[All Fields] AND "single"[All Fields] AND "nucleotide"[All Fields]) OR "single nucleotide polymorphism"[All Fields] OR "snps"[All Fields]) OR ("socioaffect neurosci psychol"[Journal] OR "snp"[All Fields]) OR (("single person"[MeSH Terms] OR ("single"[All Fields] AND "person"[All Fields]) OR "single person"[All Fields] OR "single"[All Fields] OR "singles"[All Fields]) AND ("nucleotid"[All Fields] OR "nucleotides"[MeSH Terms] OR "nucleotides"[All Fields] OR "nucleotide"[All Fields] OR "nucleotidic"[All Fields]) AND "polymorph*"[All Fields]) OR ("morphism"[All Fields] OR "morphisms"[All Fields]) OR "allele*"[All Fields] OR (("genes"[MeSH Terms] OR "genes"[All Fields] OR "gene"[All Fields]) AND ("variant"[All Fields] OR "variant s"[All Fields] OR "variants"[All Fields])) OR ("genetic predisposition to disease"[MeSH Terms] OR ("genetic"[All Fields] AND "predisposition"[All Fields] AND "disease"[All Fields]) OR "genetic predisposition to disease"[All Fields] OR ("genetic"[All Fields] AND "susceptibility"[All Fields]) OR "genetic susceptibility"[All Fields])	8,873,273
#19	Filter #18 for non-used terms, English, and human only	("gene*"[All Fields] OR "polymorphism"[All Fields] OR "single nucleotide polymorphism"[All Fields] OR "SNP"[All Fields] OR "SNP"[All Fields] OR "polymorph*"[All Fields] OR "morphism*"[All Fields] OR "allel*"[All Fields] OR "variant"[All Fields] OR "genetic predisposition"[All Fields] OR "genetic susceptibility"[All Fields]) AND ((humans[Filter]) AND (english[Filter]))	**4,633,474**
#20	#9 OR #13 OR #16	((("risk behav*"[All Fields] OR "unhealthy behav*"[All Fields] OR "eating habits"[All Fields] OR "smok*"[All Fields] OR "physical inactiv*"[All Fields] OR "alcohol*"[All Fields] OR "substance related disorders"[All Fields] OR "substance abuse"[All Fields]) AND ("humans"[MeSH Terms] AND	**1,418,464**

		"english"[Language]) AND ("humans"[MeSH Terms] AND "english"[Language])) OR (("diabetes mellitus"[All Fields] OR "diabetes"[All Fields] OR "diabet*"[All Fields] OR "insulin resistance"[All Fields]) AND ("humans"[MeSH Terms] AND "english"[Language])) OR (("cardiovascular diseases"[All Fields] OR "CVDs"[All Fields] OR "CVD"[All Fields] OR "heart diseases"[All Fields]) AND ("humans"[MeSH Terms] AND "english"[Language]))) AND ((humans[Filter]) AND (english[Filter]))	
#21	#6 & #19 & #20	(("reward pathway*"[All Fields] OR "dopamine agonists"[All Fields] OR "dopaminergics"[All Fields] OR "dopaminergic"[All Fields] OR "dopaminergically"[All Fields] OR "ventral tegmental area"[All Fields] OR "mesocorticolimbic"[All Fields] OR "mesolimbic"[All Fields] OR "corticolimbic"[All Fields]) AND ("humans"[MeSH Terms] AND "english"[Language]) AND (("gene*"[All Fields] OR "polymorphism"[All Fields] OR "single nucleotide polymorphism"[All Fields] OR "SNP"[All Fields] OR "SNP"[All Fields] OR "polymorph*"[All Fields] OR "morphism*"[All Fields] OR "allel*"[All Fields] OR "variant"[All Fields] OR "genetic predisposition"[All Fields] OR "genetic susceptibility"[All Fields]) AND ("humans"[MeSH Terms] AND "english"[Language])) AND (((("risk behav*"[All Fields] OR "unhealthy behav*"[All Fields] OR "eating habits"[All Fields] OR "smok*"[All Fields] OR "physical inactiv*"[All Fields] OR "alcohol*"[All Fields] OR "substance related disorders"[All Fields] OR "substance abuse"[All Fields]) AND ("humans"[MeSH Terms] AND "english"[Language]) AND ("humans"[MeSH Terms] AND "english"[Language])) OR (("diabetes mellitus"[All Fields] OR "diabetes"[All Fields] OR "diabet*"[All Fields] OR "insulin resistance"[All Fields]) AND ("humans"[MeSH Terms] AND "english"[Language])) OR (("cardiovascular diseases"[All Fields] OR "CVDs"[All Fields] OR "CVD"[All Fields] OR "heart diseases"[All Fields]) AND ("humans"[MeSH Terms] AND "english"[Language]))) AND ("humans"[MeSH Terms] AND "english"[Language]))) AND ((humans[Filter]) AND (english[Filter]))	**1,417**

Supplementary Materials

Table S3*:* Search strategy on Web of Science

Search	Query	Search details	Number of hits
#1	Mesocorticolimbic	ALL=("reward pathway*" OR "dopamine agonists" OR "dopaminergics" OR "dopaminergic" OR "dopaminergically" OR "ventral tegmental area" OR "mesocorticolimbic" OR "mesolimbic" OR "corticolimbic")	90,316
#2	Cardiovascular diseases	ALL=("cardiovascular diseases" OR "CVDs" OR "CVD" OR "heart diseases")	186,125
#3	Diabetes Mellitus	ALL=("diabetes mellitus" OR "diabetes" OR "diabet*" OR "insulin resistance")	1,366,127
#4	Risk behaviours	ALL=("risk behav*" OR "unhealthy behav*" OR "eating habits" OR "smok*" OR "physical inactiv*" OR "alcohol*" OR "substance related disorders" OR "substance abuse")	1,120,148
#5	Genetic variants	ALL=("gene*" OR "polymorphism" OR "single nucleotide polymorphism" OR "SNP" OR "snp" OR "polymorph*" OR "morphism*" OR "allel*" OR "variant" OR "genetic predisposition" OR "genetic susceptibility")	14,110,884
#6	Combine CVDs, DM, and risk behaviours	#2 OR #3 OR #4	2,553,897
#7	#1 & #5 & #6		3,318
#8	Filter #7 for English and remove reviews	#2 OR #3 OR #4 (Exclude – Document Types) and English (Languages)	**2,250**

Supplementary Materials

Table S4: Gene Catalog: Chromosome Assignment and Functional Roles

Gene	Chromosome	Role
5HT2A/5HT2AR	13	Serotonin receptor 2A
5HT2C	X	Serotonin receptor 2C
ADH1B	4	Alcohol dehydrogenase 1B
ADRA2A	10	Adrenoceptor alpha 2A
AGBL4	1	ATP/GTP binding protein-like 4
ANKK1	11	Ankyrin repeat and kinase domain 1
ANKS1B	10	Ankyrin repeat and sterile alpha motif
ANNK1	11	Ankyrin repeat and kinase domain 1
AP2A2	15	Adaptor-related protein complex 2
BZRAP1	7	Benzodiazepine receptor-associated protein 1
CDNF	1	Cerebral dopamine neurotrophic factor
CHRM5	15	Muscarinic acetylcholine receptor 5
CHRNA3	15	Nicotinic acetylcholine receptor alpha 3
CHRNA4	20	Nicotinic acetylcholine receptor alpha 4
CHRNA5	15	Nicotinic acetylcholine receptor alpha 5
CHRNA6	8	Nicotinic acetylcholine receptor alpha 6
CHRNB2	1	Nicotinic acetylcholine receptor beta 2
CHRNB3	8	Nicotinic acetylcholine receptor beta 3
CNTFR	9	Ciliary neurotrophic factor receptor
COMT	22	Catechol-O-methyltransferase
CRH	8	Corticotropin-releasing hormone
CSNK1E	22	Casein kinase 1 epsilon
CTNNA2	2	Catenin alpha 2
DAT1	5	Dopamine transporter 1
DBH	9	Dopamine beta-hydroxylase
DCC	18	Deleted in colorectal cancer
DDC	7	Dopadecarboxylase
DLG2	11	Discs large MAGUK scaffold protein 2
DRD1	5	Dopamine receptor D1
DRD2	11	Dopamine receptor D2
DRD3	3	Dopamine receptor D3
DRD4	11	Dopamine receptor D4
DRD5	4	Dopamine receptor D5
DRD5	1	Dopamine receptor D5 (alternative form)
DβH	9	Dopamine beta-hydroxylase
FAAH	1	Fatty acid amide hydrolase
FAT3	11	FAT atypical cadherin 3
FIGNL1	12	Fidgetin-like 1
GABBR2	9	Gamma-aminobutyric acid B receptor 2
GALR1	18	Galanin receptor 1
GHRL	3	Ghrelin precursor
GHSR	3	Growth hormone secretagogue receptor

Supplementary Materials

GLP1R	6	Glucagon-like peptide-1 receptor
GRB10	7	Growth factor receptor-bound protein 10
GRIK1	21	Glutamate receptor ionotropic kainate 1
HTR1A	5	5-hydroxytryptamine receptor 1A
HTR1B	6	5-hydroxytryptamine receptor 1B
HTR1E	6	5-hydroxytryptamine receptor 1E
HTR2A	13	5-hydroxytryptamine receptor 2A
HTR2A	16	5-hydroxytryptamine receptor 2A (alternative form)
KTN1	14	Kinectin 1
LEPR	1	Leptin receptor
MAOA	X	Monoamine oxidase A
MAP3K4	6	Mitogen-activated protein kinase kinase kinase 4
MC4R	18	Melanocortin 4 receptor
NCAM1	11	Neural cell adhesion molecule 1
NGFR	17	Nerve growth factor receptor
NTF3	12	Neurotrophin 3
NTRK2	9	Neurotrophic tyrosine kinase receptor 2
NTRK3	15	Neurotrophic tyrosine kinase receptor 3
OPRD	1	Delta-type opioid receptor
OPRM1	6	Mu-type opioid receptor
PIP4K2A	10	Phosphatidylinositol 5-phosphate 4-kinase type 2 alpha
PPP1R1B	17	Protein phosphatase 1 regulatory inhibitor subunit 1B
SERT	17	Serotonin transporter
SLC6A3	5	Sodium-dependent dopamine transporter
SLC6A4	17	Serotonin transporter
SPR 2	2	Sepiapterin reductase
TH	11	Tyrosine hydroxylase
TPH2	12	Tryptophan hydroxylase 2
TTC12	8	Tetratricopeptide repeat domain 12
VMAT2	10	Vesicular monoamine transporter 2
ZNFN1A1	4	Zinc finger protein N1A1

Supplementary Materials

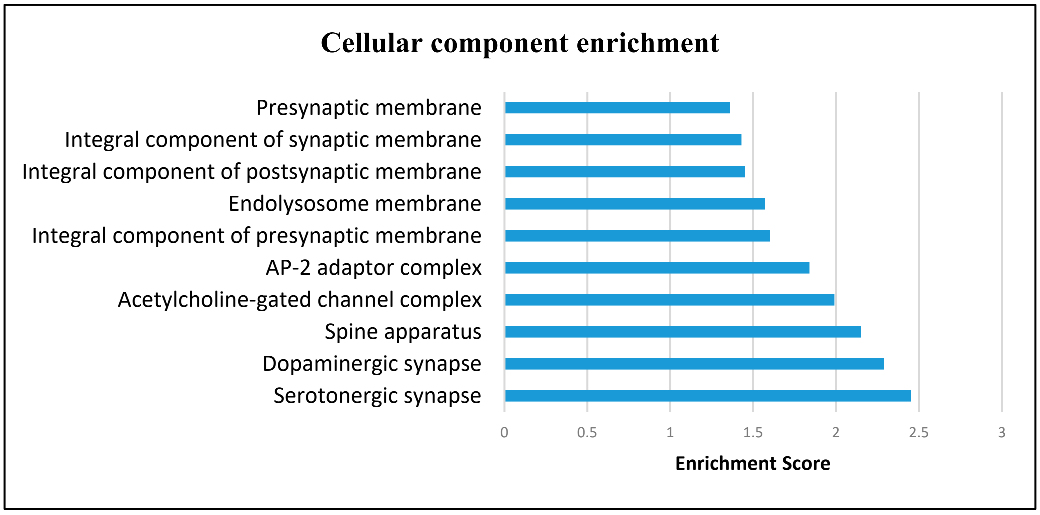

Figure S1*:* The top ten cellular component enrichment terms of the identified genes

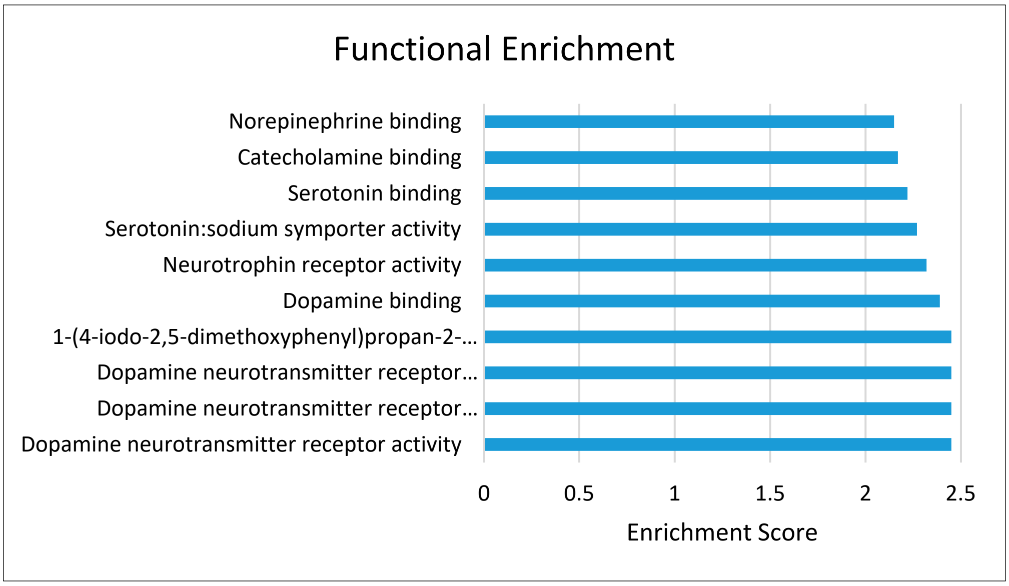

Figure S2*:* The top ten functional enrichment terms of the identified genes

Supplementary Materials

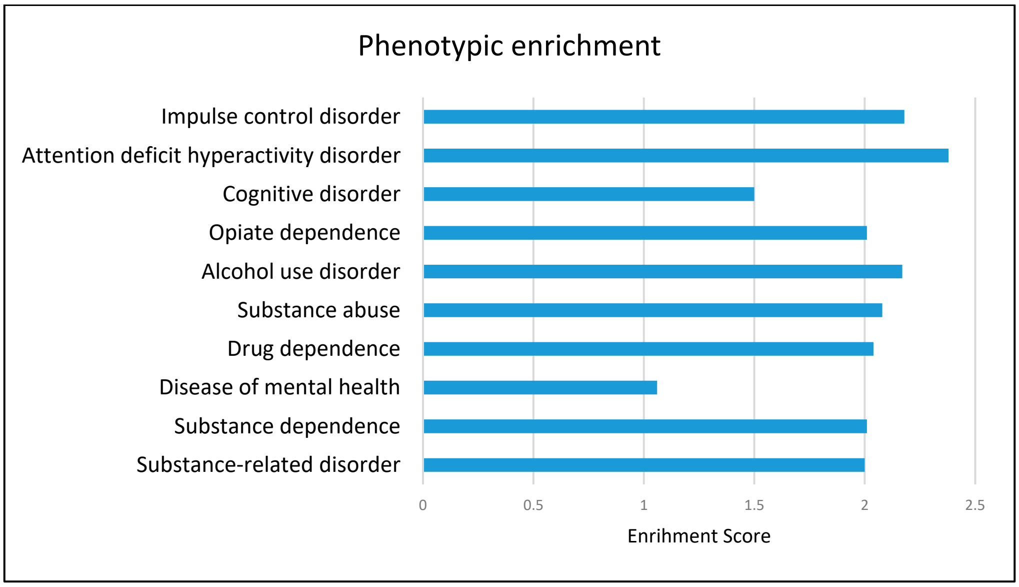

Figure S3: The top ten phenotypic enrichment terms of the identified genes
